# Supplementary material for: Deep-branching Chloroflexota lineages illuminate the eco-evolutionary foundation of cross-ecosystem colonization
Source: Nat Commun. 2026 Apr 1;17:4696. doi: 10.1038/s41467-026-71228-y (PMC13212565; doi:10.1038/s41467-026-71228-y)
Supplement: Supplementary file 1 — Supplementary Information [file 41467_2026_71228_MOESM1_ESM.pdf]

Supplementary material

Deep-branching *Chloroflexota* lineages illuminate the eco-evolutionary foundation of cross-ecosystem colonization

Lucas Serra Moncadas[1], Alisa Shakurova[1], Cyrill Hofer[1], Adrian-Stefan Andrei[1,*]

[1]Limnological Station, Department of Plant and Microbial Biology, University of Zurich; Kilchberg 8802, Switzerland.

*Corresponding author. Email: stefan.andrei@limnol.uzh.ch

Supplementary Note 1

Genome-resolved metabolic reconstruction was utilized to gain key insights into the ecological strategies and metabolic capabilities of the analyzed Chloroflexota families, specifically P2-11E, QHBO01, CSP1-4, and Limnocylindraceae. Core metabolic functions, including carbohydrate, energy, and amino acid metabolism, were examined alongside non-core ones such as transporters, sensing, and motility mechanisms.

Transporters

Transporters act as the primary interface between bacteria and their surrounding environment, facilitating the uptake of organic and inorganic compounds available within their ecological niche. All analyzed Chloroflexota families displayed diverse transporters (Supp. Data S6), with distinct preferences that reflect habitat specificities.

The plant-derived polysaccharide transport systems were found to be enriched in the soil-associated lineages P2-11E, QHBO01, and CSP1-4. These systems include the raffinose/stachyose/melibiose transport system substrate-binding protein (msmE), permease protein (msmF), D-xylose transport system substrate-binding protein (xylF), permease protein (xylH), ATP-binding protein (xylG), and polysaccharide transporter PST family (TC.PST). The genomic densities for these transporters were measured at 0.90, 1.33, and 1.57 transporters/Mb for P2-11E, QHBO01, and CSP1-4, respectively. Notably, these transport systems were found absent in the freshwater Limnocylindraceae lineage.

Metabolism

Plant-derived polysaccharides, such as D-xylose, are likely metabolized via a series of enzymatic reactions that ultimately fuel essential cellular processes. Thus, D-xylose can be first isomerized by xylose isomerase to form D-xylulose, which is subsequently phosphorylated by xylulokinase into D-xylulose-5-phosphate (xylulose-5P). This compound may be further epimerized by ribulose-phosphate 3-epimerase to yield D-ribulose-5P. This set of enzymes was found present among all the soil-associated lineages including P2-11E (n = 15), QHBO01 (n = 5), CSP1-4 (n = 42) and absent in the freshwater lineage (i.e., Limnocylindraceae). This substrate then may enter the Pentose Phosphate Pathway (PPP)—found to be complete across all Chloroflexota families (Supp. Data S9).

Within the PPP, D-ribulose-5P could be isomerized by ribose 5-phosphate isomerase B into D-ribose-5P. A transketolase could then transfer two carbons from D-xylulose-5P to D-ribose-5P, producing D-glyceraldehyde-3-phosphate (D-G3P). Families P2-11E (n = 30), QHBO01 (n =6), CSP1-4 (n = 73) and Limnocylindraceae

(n = 53) all exhibited this complete set of enzymes. The soil-associated lineages (i.e., P2-11E, QHBO01, and CSP1-4) were found to possess the xylulose-5-phosphate/fructose-6-phosphate phosphoketolase enzyme, which can cleave D-xylulose-5P into D-G3P. The key metabolite D-G3P could further enter the glycolysis core module, where it can undergo several enzymatic transformations to generate pyruvate. Thus, D-G3P can be oxidized and phosphorylated by glyceraldehyde-3-phosphate dehydrogenase, forming D-glycerate-1,3-diphosphate. This intermediate may be further dephosphorylated by phosphoglycerate kinase to yield D-glycerate-3P, which can subsequently be converted into D-glycerate-2P by 2,3-bisphosphoglycerate-independent phosphoglycerate mutase. The subsequent step in glycolysis involves the dehydration of D-glycerate-2P by enolase, yielding phosphoenolpyruvate (PEP). PEP may further be converted into pyruvate by the pyruvate kinase. The entire set of enzymes performing these reactions was found complete among the soil-related lineages, P2-11E (n = 15), QHBO01 (n = 6) and CSP1-4 (n = 41). Pyruvate serves as a central metabolite that can be further oxidized to acetyl-CoA, thereby fueling the Tricarboxylic Acid (TCA) cycle and aerobic respiration or acting as a precursor for the biosynthesis of macromolecules. Among the analyzed Chloroflexota families, a complete pyruvate oxidation pathway (POP) and TCA cycle were identified in the P2-11E (POP n = 29 ; TCA n = 13), QHBO01 (POP n = 6; TCA n = 6), CSP1-4 (POP n = 72; TCA n = 48), and Limnocylindraceae (POP n = 53; TCA n = 63) lineages.

In the context of oxidative phosphorylation, all studied Chloroflexota families possessed complete gene sets encoding for the Complex I (NuoA-NuoN), Complex II (SdhC, SdhD, SdhA, SdhB), Complex IV (CoxD, CoxC, CoxA, CoxB), and Complex V (F-type ATPase). The presence of these fully assembled respiratory gene complexes strongly indicates that these families (P2-11E n = 38, QHBO01 n = 8, CSP1-4 n = 62, Limnocylindraceae n = 60) are adapted to an aerobic lifestyle, facilitating efficient electron transport and ATP synthesis via oxidative phosphorylation.

Analysis of proteinogenic amino acid biosynthetic pathways revealed a significant discrepancy between terrestrial and freshwater Chloroflexota lineages. Specifically, the P2-11E, QHBO01, and CSP1-4 families possess the necessary machinery to synthesize the majority of proteinogenic amino acids (Supp. Data S7), whereas the Limnocylindraceae exhibit a reduced capacity, being capable of synthesizing only eight of the twenty standard amino acids.

Remarkably, the metabolic architecture of the CSP1-4 family exhibits a distinctive combination of terrestrial and aquatic adaptations. Specifically, the presence of plant-related polysaccharide transporters indicates terrestrial affinities, while their flagellar apparatus points toward aquatic adaptation. Surprisingly, this clade encodes the complete panoply of genes necessary for flagellar assembly (n = 15),

including fliE, fliF, fliG, fliH, fliJ, fliK, fliL, fliM, fliN, flgA, flgB, flgC, flgD, flgE, flgF, flgH, flgI, and flgJ. Additionally, genes responsible for chemotaxis were identified (n = 32) within CSP1-4, suggesting that members of this clade are likely motile. Although the presence of flagella may appear to be a minor trait, it is notably uncommon among Chloroflexota members, with only the genus Tepidiforma previously reported to exhibit this characteristic[1].

While soil-related Chloroflexota families (i.e., P2-11E, QHBO01, and CSP1-4) appear to possess optimized tools for exploiting resources in terrestrial environments—with CSP1-4 serving as an intermediate lineage bridging soil and freshwater adaptations—their freshwater relative, Limnocylindraceae, exhibits significant differences. Thus, Limnocylindraceae shows an increase in amino acid transporters, accounting for an average of 6.2 transporters/Mb, compared to its soil relatives CSP1-4 (3.6 transporters/Mb) and QHBO01 (3.8 transporters/Mb). Imported amino acids likely provide Limnocylindraceae with essential building blocks, as this lineage suffers from severe impairments in amino acid biosynthetic pathways, and also serve as a source of energy through amino acid catabolism. For instance, aspartate can be catalyzed by adenylosuccinate synthase in combination with adenylosuccinate lyase to form fumarate, which could further enter the TCA cycle. Both of these enzymes were found present in Limnocylindraceae (n = 50). Alternatively, aspartate can be transaminated to form oxaloacetate via aspartate aminotransferase. A more direct pathway involves the deamination of serine to pyruvate by serine dehydratase and the conversion of threonine to α-ketobutyrate by threonine dehydratase. Both of serine and threonine dehydratase were consistently detected in Limnocylindraceae (n = 79; n = 79) As previously mentioned, pyruvate serves as a substrate for pyruvate oxidation, generating acetyl-CoA, which in turn can fuel aerobic respiration.

Another distinguishing feature of the Limnocylindraceae clade is the presence of rhodopsins (n = 65), specifically bacteriorhodopsins (bop), which are absent in the soil-related Chloroflexota lineages (P2-11E, QHBO01, and CSP1-4). Rhodopsins are photoreceptive membrane proteins capable of sensing and responding to light signals. They perform a variety of functions, from mediating phototaxis through photosensory rhodopsins to generating energy via light-driven proton (H^+) pumps[2]. In the case of Limnocylindraceae, bacteriorhodopsins likely convert light energy into proton gradients across the membrane. These gradients are further utilized by ATP synthase (ATPase) to produce ATP, thereby supplying cellular energy. This mechanism is particularly advantageous in aquatic environments, where optimal light penetration facilitates the efficient utilization of rhodopsins.

Supplementary Note 2

To trace how functional traits evolved in response to major ecosystem shifts, we performed ancestral state reconstructions across the Chloroflexota phylogeny using discrete-character models (see Methods). The results reveal that key metabolic and sensory capacities were independently acquired or lost along multiple lineages, echoing genome-wide trends observed in metabolic reconstructions.

In the Limnocylindraceae family, bacteriorhodopsins were inferred to be absent from the most recent common ancestor (MRCA) but emerged with high posterior support in derived freshwater clades (Supp. Fig. S14), suggesting phototrophic potential was gained following the transition from terrestrial to aquatic niches. Similarly, genes encoding flagellar motility and chemotaxis modules were reconstructed as absent in the MRCA of the CSP1-4 family, yet acquired deeper in the lineage—likely reflecting increased spatial heterogeneity and resource patchiness in freshwater environments. The co-occurrence of soil- and water-adapted traits in extant CSP1-4 genomes further supports a dual ecological signature shaped by niche expansion.

In contrast, traits such as the heme biosynthesis pathway and multiple amino acid biosynthetic modules (e.g., for histidine, methionine, lysine, and tryptophan) were reconstructed as present in the Limnocylindraceae MRCA but lost in extant freshwater-adapted genomes. These convergent losses point to a major reductive episode in genome evolution, consistent with observed patterns of gene loss and elevated mutation pressure (see Figs. 3,5; Supp. Fig. S14).

Together, these reconstructions illuminate how cross-ecosystem transitions reshaped genome content through a mosaic of trait acquisition and functional erosion—linking niche shifts to the long-term architectural reconfiguration in free-living bacterial genomes.

Supp. Table S1. Summary of positive (diversifying) selection in Limnocylindria. The table presents the percentage of genes identified as being under positive selection, as well as the median number of positively selected sites per gene.

Lineage	[%] proteins under positive selection	Median sites/protein under positive selection
P2-11E	19.878	1.3
QHBO01	20.052	1.207
CSP1-4	32.113	1.514
Limnocylindraceae	17.654	1.25

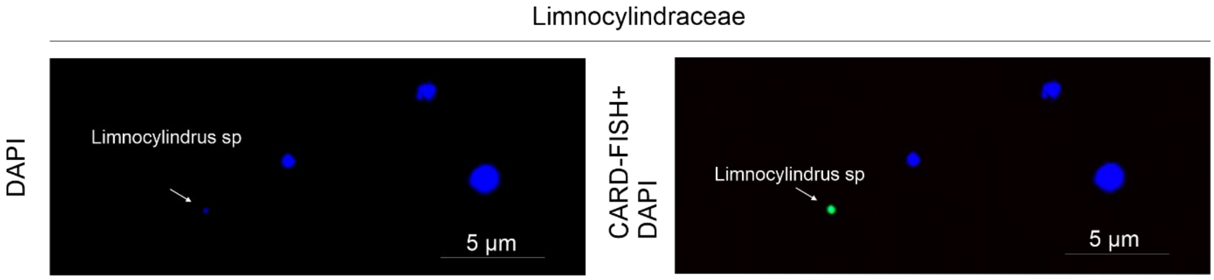

Supp. Fig. S1. Limnocylindrus cells in Lake Zurich water. DAPI-stained prokaryotes overlaid with a CARD-FISH-stained image of Limnocylindrus bacteria from Lake Zurich. Each panel is a single optical section; brightness and contrast were adjusted for both images using imaging software.

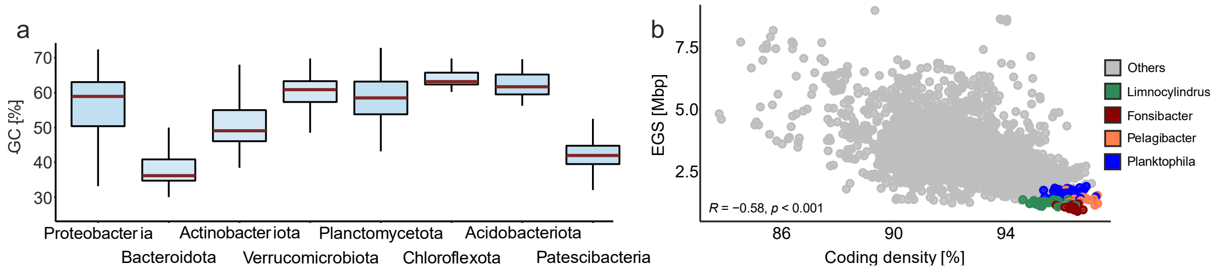

Supp. Fig. S2. PdCEL genomic properties. (a) GC content variations across the eight dominant bacterial phyla found in the pdCEL database. Each boxplot delineates the interquartile range, spanning from the first quartile (25th percentile) to the third quartile (75th percentile) and encompassing the central 50% of the data. The whiskers extend from the boxes to the most extreme data points that are not classified as outliers, illustrating the overall spread of the main dataset. Plot (b) illustrates the estimated genome size (EGS) and coding density relationship assessed through Pearson's correlation test. Small genome representatives (i.e., Limnocylindraceae, Fonsibacter, Pelagibacter, and Planktophila) are highlighted with four distinctive colors. Source data are provided as a Source Data file.

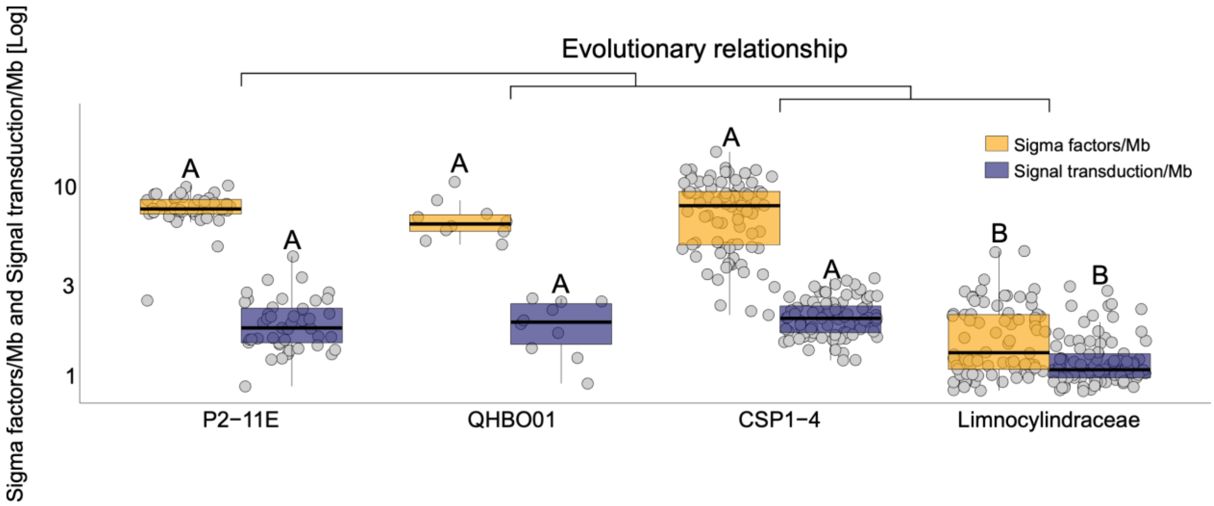

Supp. Fig. S3. Dynamics of sigma factors and signal transduction domains. The figure illustrates the normalized number of sigma factors and signal transduction domains per megabase pair (Mb) across the major evolutionary lineages of Limnocylindria. The X-axis represents the taxonomic categories, while the Y-axis indicates the normalized abundance. Uppercase letters positioned above the boxplots signify different statistical categories, highlighting significant differences between groups. Boxplots sharing the same capital letter indicate no significant difference ($p > 0.01$), while boxplots with different capital letters are significantly different ($p \leq 0.01$). Each boxplot delineates the interquartile range, spanning from the first quartile (25th percentile) to the third quartile (75th percentile) and encompassing the central 50% of the data. The whiskers extend from the boxes to the most extreme data points that are not classified as outliers, illustrating the overall spread of the main dataset. All supplementary statistics are reported in Supp. Data S15. Source data are provided as a Source Data file.

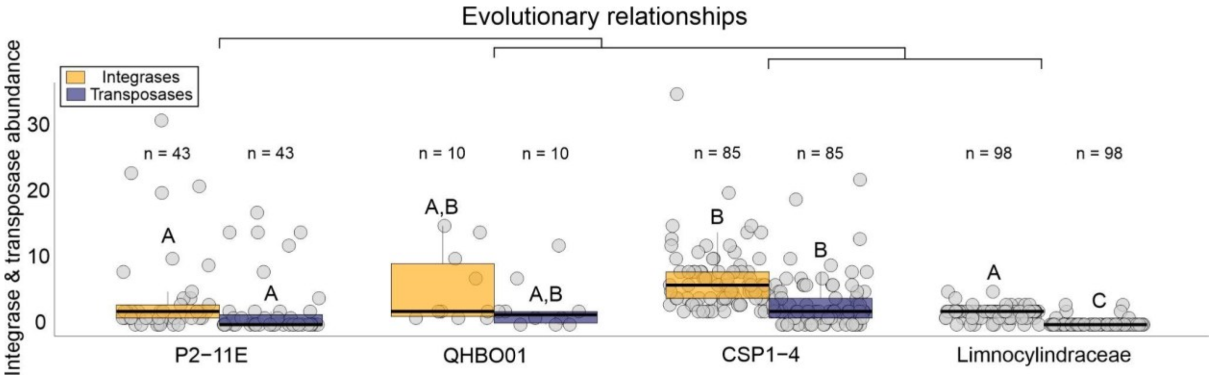

Supp. Fig. S4. Integrase and transposase dynamics. The figure depicts the abundance of integrases (orange) and transposases (purple) across the Limnocylindria lineages. The X-axis represents the taxonomic categories, while the Y-axis indicates the abundance. Uppercase letters positioned above the boxplots signify different statistical categories, highlighting significant differences between groups. Boxplots sharing the same capital letter indicate no significant difference ($p > 0.01$), while boxplots with different capital letters are significantly different ($p \leq 0.01$). Each boxplot delineates the interquartile range, spanning from the first quartile (25th percentile) to the third quartile (75th percentile) and encompassing the central 50% of the data. The whiskers extend from the boxes to the most extreme data points that are not classified as outliers, illustrating the overall spread of the main dataset. All supplementary statistics are reported in Supp. Data S15. Source data are provided as a Source Data file.

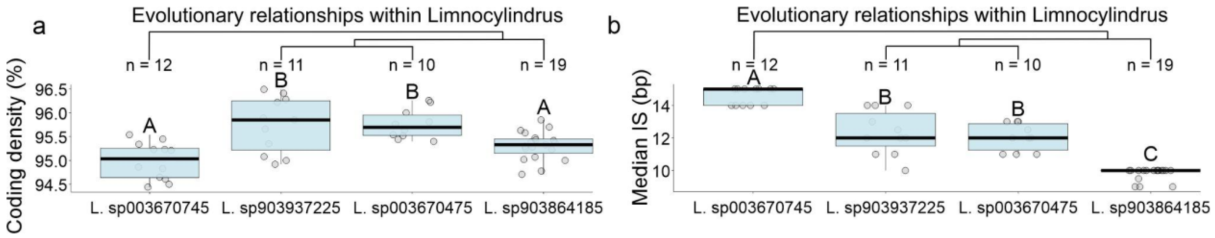

Supp. Fig. S5. **Distribution of coding density and intergenic spacer length within Limnocylindrus**. The X-axis designates taxonomic categories, while the Y-one absolute values. a) Coding density for Limnocylindrus species. b) Median intergenic spacer in base pair length for Limnocylindrus species. Uppercase letters positioned above the boxplots signify different statistical categories, highlighting significant differences between groups. Boxplots sharing the same capital letter indicate no significant difference ($p > 0.05$), while boxplots with different capital letters are significantly different ($p \leq 0.05$). Each boxplot delineates the interquartile range, spanning from the first quartile (25th percentile) to the third quartile (75th percentile) and encompassing the central 50% of the data. The whiskers extend from the boxes to the most extreme data points that are not classified as outliers, illustrating the overall spread of the main dataset. All supplementary statistics are reported in Supp. Data S15. Source data are provided as a Source Data file.

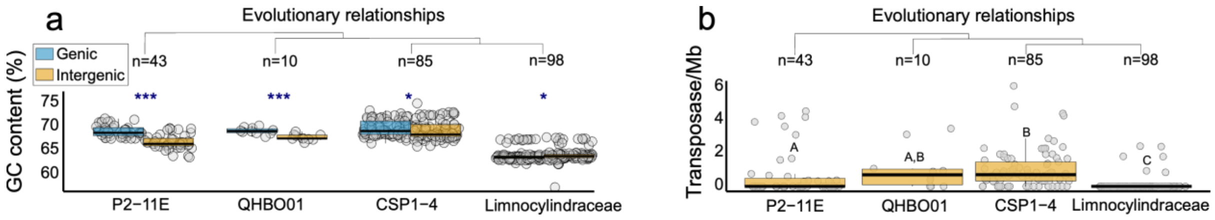

Supp. Fig. S6.GC content and mobile genetic elements distribution. (a) GC content distribution between genic and intergenic genomic regions in Limnocylindria lineages. Stars above the boxplots indicate statistically significant differences between the two categories. (b) Number of transposases in Limnocylindria lineages adjusted for genome size. Pairwise statistical differences are highlighted via the means of capital letters and stars (*$p < 0.05$; **$p < 0.01$; ***$p < 0.001$, ns ≤ 0.05). Boxplots sharing the same capital letter indicate no significant difference ($p > 0.05$), while boxplots with different capital letters are significantly different ($p \leq 0.05$). Each boxplot represents the interquartile range, spanning from the first quartile (25th percentile) to the third quartile (75th percentile), thereby encompassing the central 50% of the data. Whiskers extend from the boxes to the most extreme non-outlier data points, illustrating the overall spread of the dataset. All supplementary statistics are reported in Supp. Data S15. Source data are provided as a Source Data file.

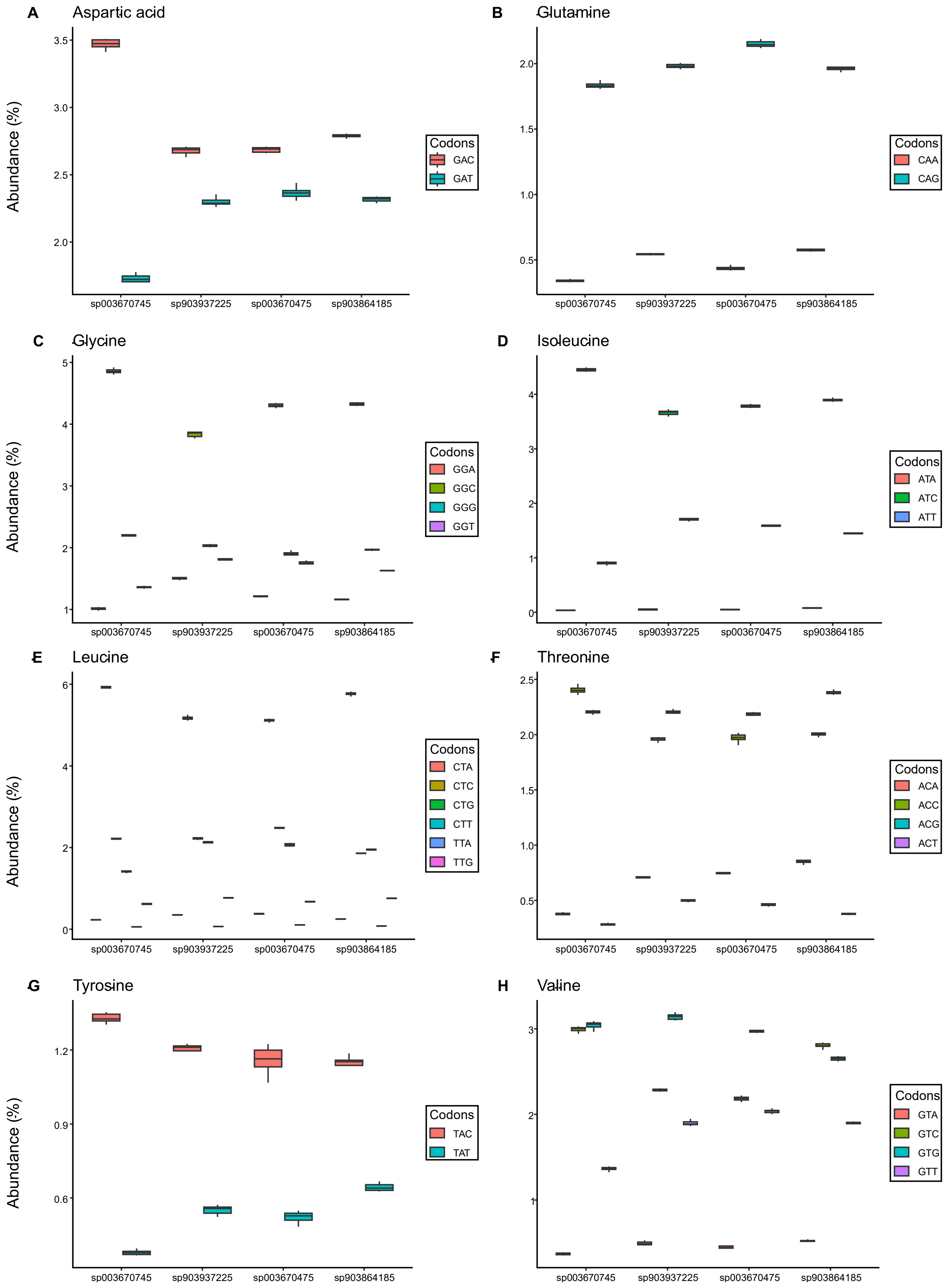

Supp. Fig. S7. Amino acid codon frequencies at the species level. Panels (A-H) represent codon frequency distributions for various Limnocylindraceae species (*L. sp003670745*; *L. sp903937225*; *L. sp003670475*; *L. sp903864185*). The Y-axis displays codon frequencies as a percentage, while the X-axis the respective

taxonomic labels ordered phylogenetically. Colours represent specific codons used to synthesize each amino acid. The central line across the boxplots identifies the median, marking the dataset's midpoint. The box itself demarcates the interquartile range, extending from the first quartile to the third quartile, encapsulating the central 50% of the data. The whiskers project from the box to the furthest data points not categorized as outliers and show the spread of the main body of the dataset. Source data are provided as a Source Data file.

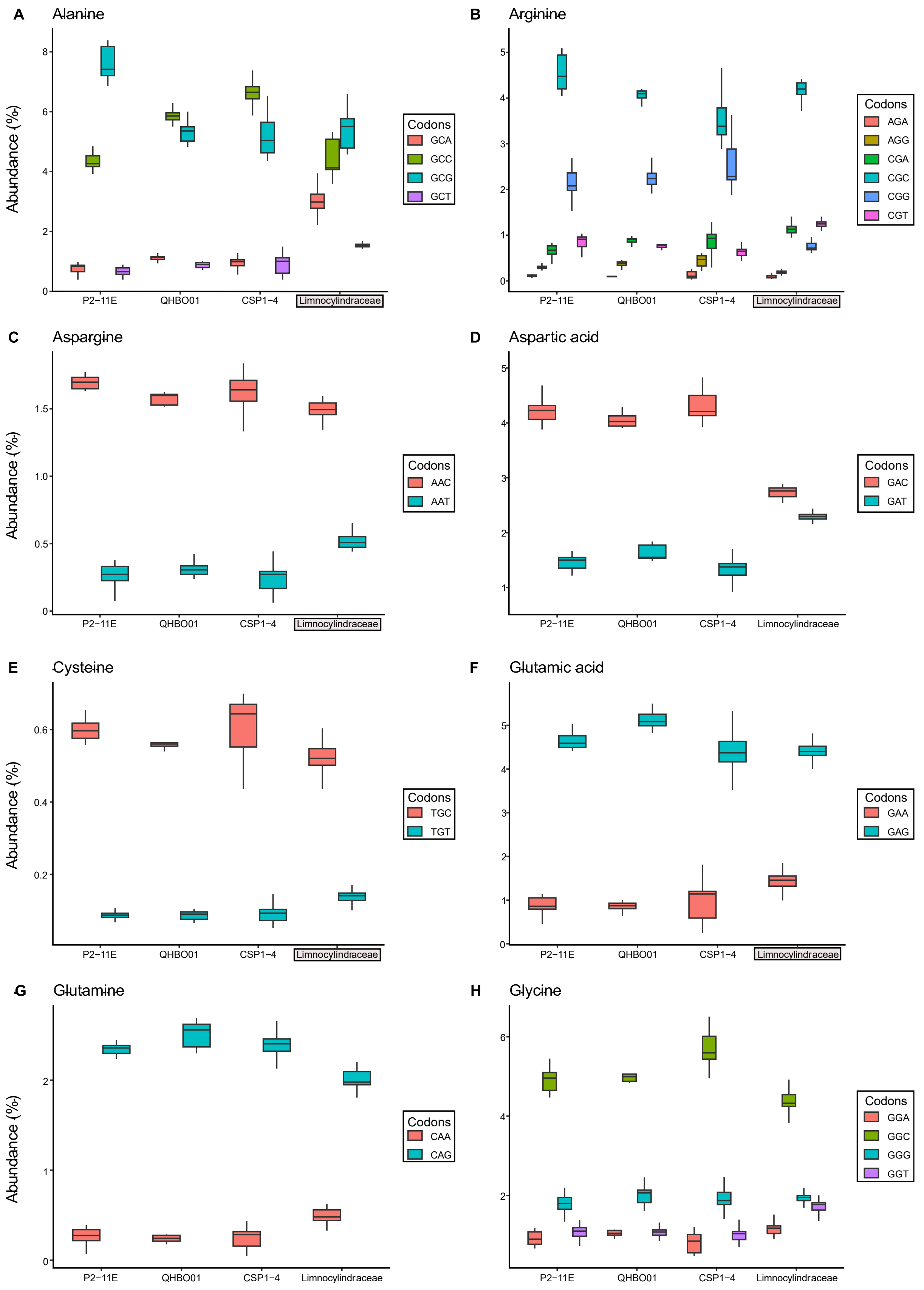
A
Alanine
B
Arginine
C
Aspargine
D
Aspartic acid
E
Cysteine
F
Glutamic acid
G
Glutamine
H
Glycine
Abundance (%)
P2−11E
QHBO01
CSP1−4
Limnocylindraceae
Codons
GCA
GCC
GCG
GCT
AGA
AGG
CGA
CGC
CGG
CGT
AAC
AAT
GAC
GAT
TGC
TGT
GAA
GAG
CAA
CAG
GGA
GGC
GGG
GGT

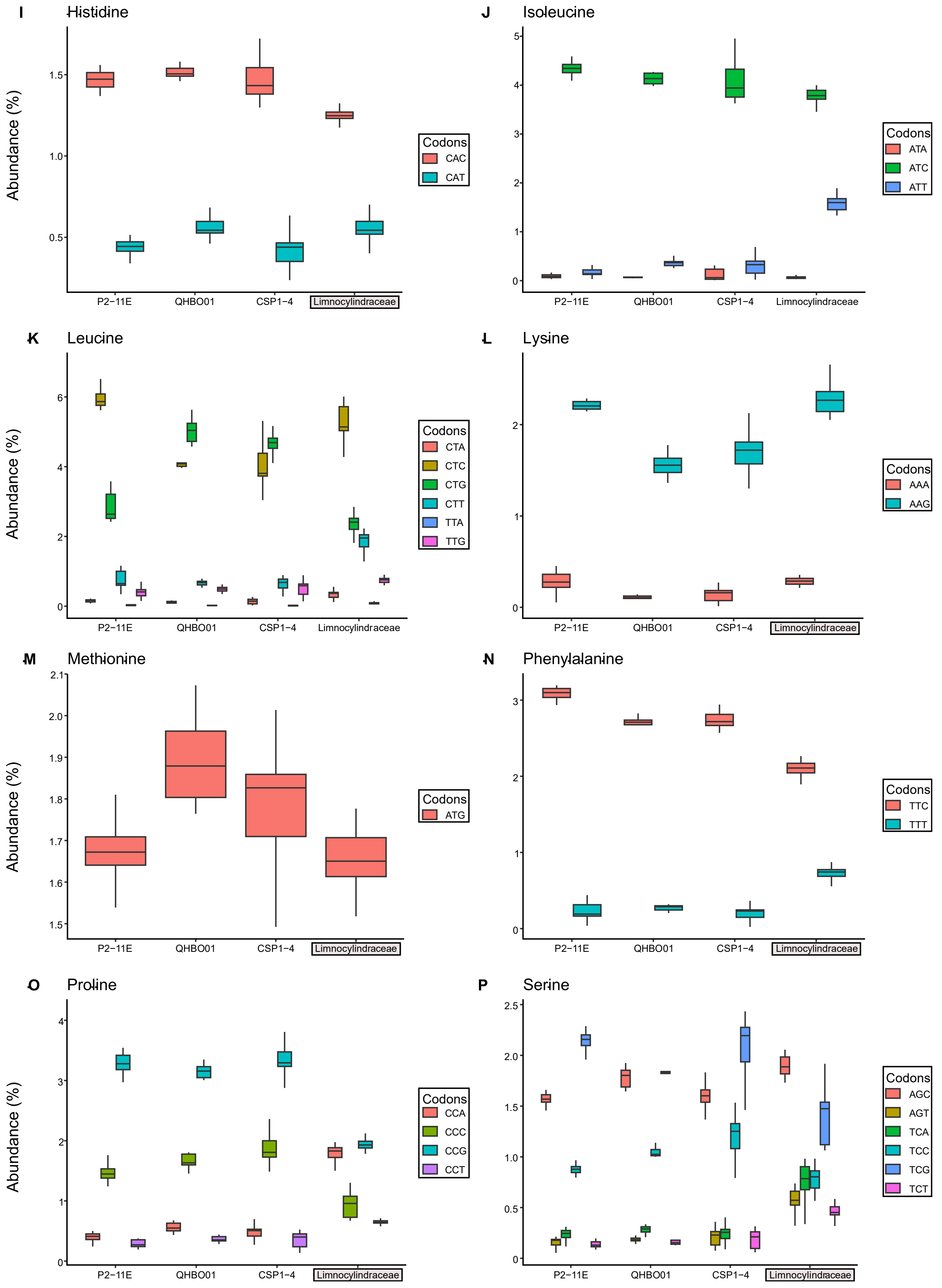
I
Histidine
Abundance (%)
Codons
CAC
CAT
P2−11E
QHBO01
CSP1−4
Limnocylindraceae
J
Isoleucine
Codons
ATA
ATC
ATT
K
Leucine
Codons
CTA
CTC
CTG
CTT
TTA
TTG
L
Lysine
Codons
AAA
AAG
M
Methionine
Codons
ATG
N
Phenylalanine
Codons
TTC
TTT
O
Proline
Codons
CCA
CCC
CCG
CCT
P
Serine
Codons
AGC
AGT
TCA
TCC
TCG
TCT

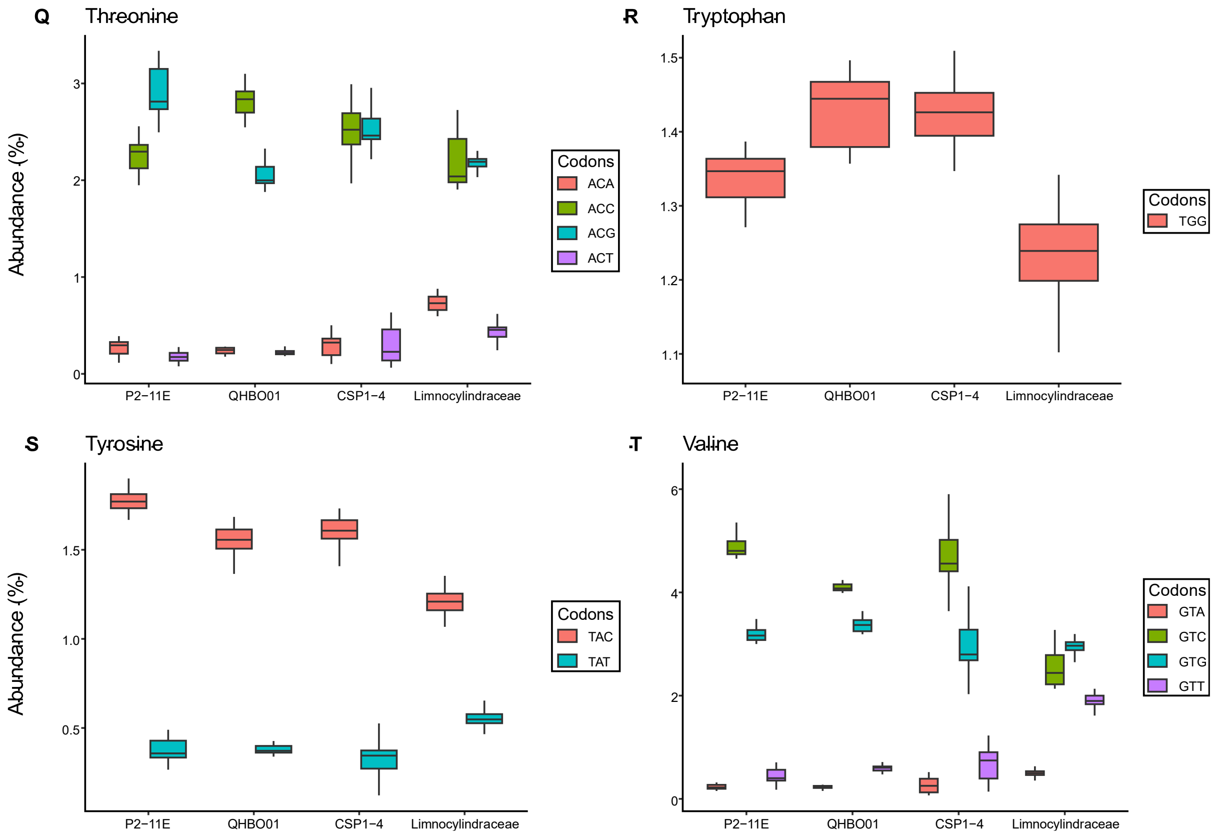

Supp. Fig. S8. Amino acid codon frequencies at the family level. Panels (A–T) display codon frequency distributions for various Limnocylindria families (P2-11E; QHBO01; CSP1-4; Limnocylindraceae). The Y-axis represents codon frequencies as a percentage, while the X-axis denotes the respective taxonomic labels ordered in a phylogenetic fashion. The grey box highlights the cases where the Limnocylindraceae family is unable to synthesize the corresponding amino acid. Colours represent the specific codons used to synthesize each amino acid. The central line across the boxplots identifies the median, marking the dataset's midpoint. The box itself demarcates the interquartile range, extending from the first quartile to the third quartile, encapsulating the central 50% of the data. The whiskers project from the box to the furthest data points not categorized as outliers and show the spread of the main body of the dataset. Source data are provided as a Source Data file.

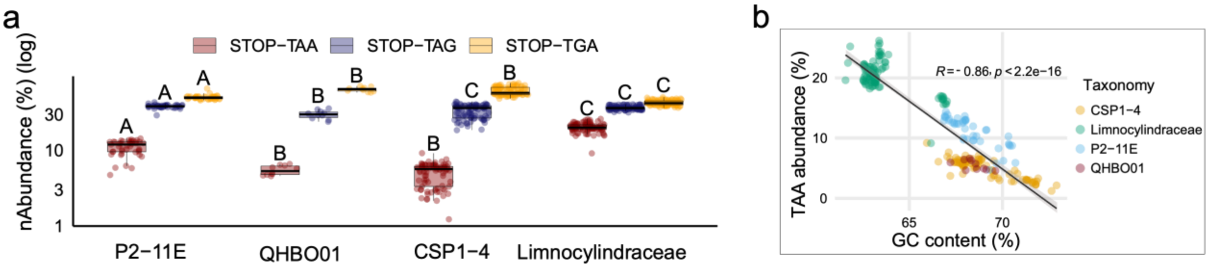

Supp. Fig. S9. Stop codon dynamics in Limnocylindraceae. (a) Stop codon normalized abundance (nAbundance) in Limnocylindria. Colors indicate the different stop codons. Uppercase letters positioned above the boxplots denote statistical categories, highlighting significant differences between groups. Boxplots sharing the same capital letter indicate no significant difference ($p > 0.05$), while boxplots with different capital letters are significantly different ($p \leq 0.05$). Each boxplot represents the interquartile range, spanning from the first quartile (25th percentile) to the third quartile (75th percentile), thereby encompassing the central 50% of the data. Whiskers extend from the boxes to the most extreme non-outlier data points, representing the overall spread of the dataset. (b) Linear relationship between the abundance of the TAA stop codon and GC content in Limnocylindria. The grey bands represent 95% confidence interval for the mean predicted by the regression. All supplementary statistics are reported in Supp. Data S15. Source data are provided as a Source Data file.

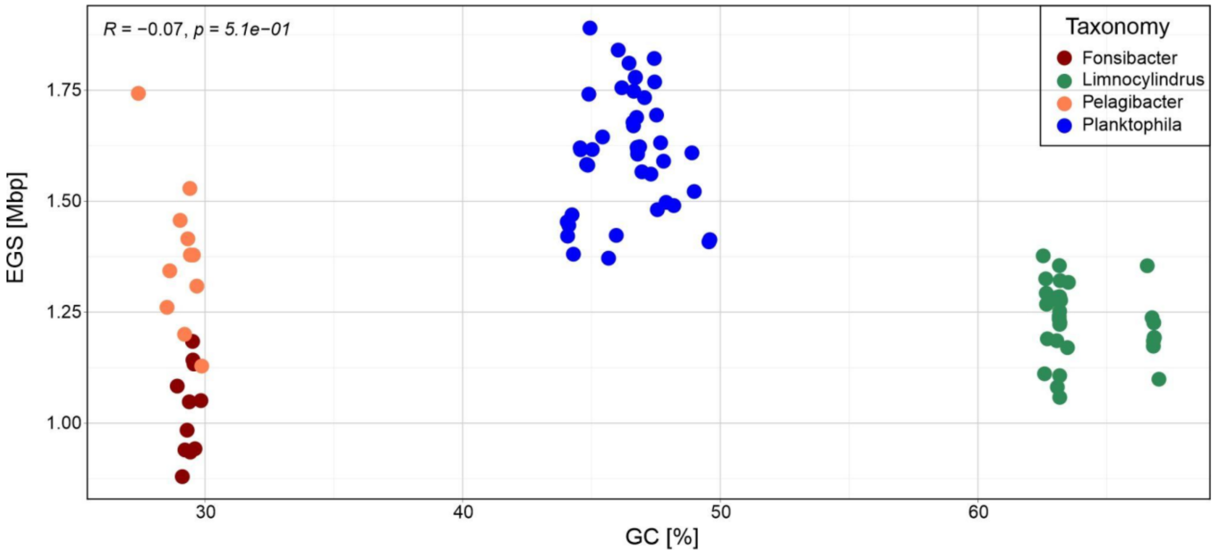

Supp. Fig. S10. Relationship between estimated genome size (EGS) and GC content. The Y-axis represents the estimated genome size, while the X-axis represents the GC content. Reduced genome taxa are shown with four different colours. Source data are provided as a Source Data file.

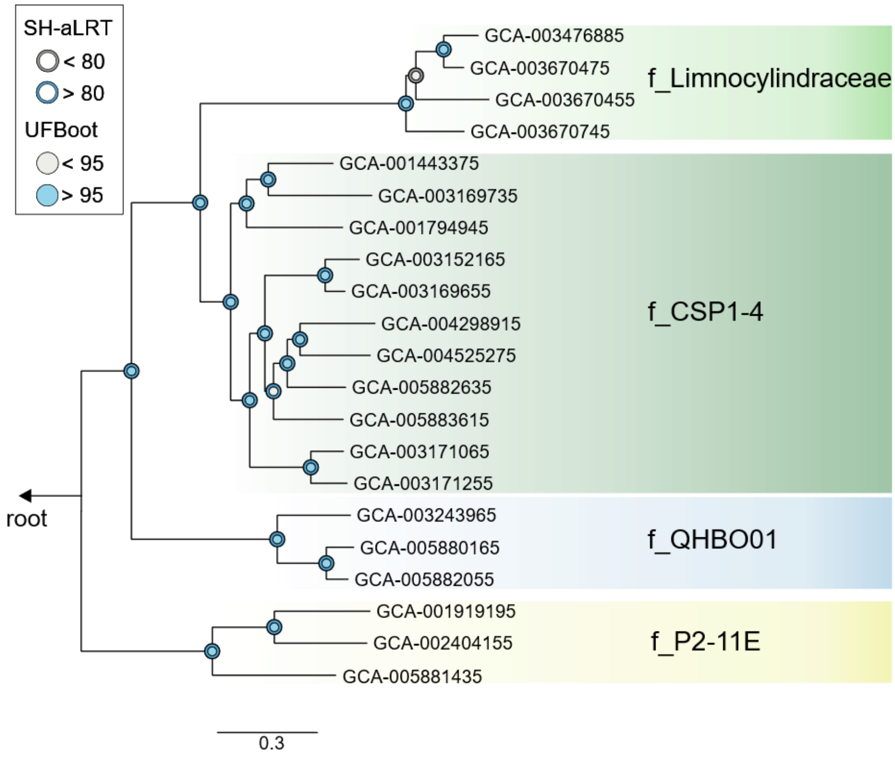

Supp. Fig. S11. Jack-knifed family-centric genome-based phylogeny. Evolutionary history of the Limnocylindria class (Chloroflexota phylum) generated through maximum-likelihood phylogeny (-m LG+C30+F+R10). Blue rings and circles denote SH-aLRT and UFBoot support values exceeding 80 and 95, respectively. The scale bar indicates the number of substitutions per site. Outgroup rooting was performed by using members of the Eremiobacterota phylum. Source data are provided as a Source Data file.

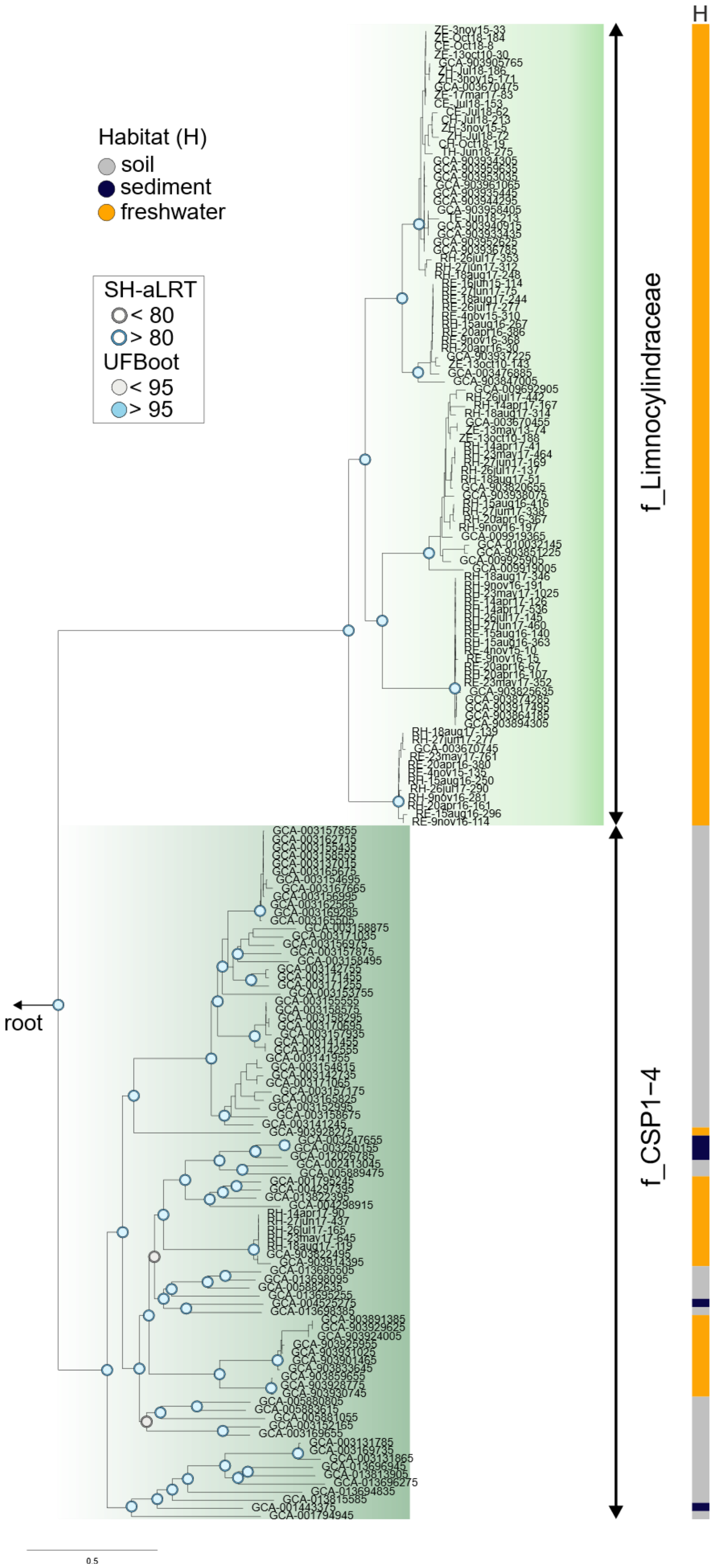

Habitat (H)
soil
sediment
freshwater
SH-aLRT
< 80
> 80
UFBoot
< 95
> 95
root
f_Limnocylindraceae
f_CSP1–4
H
0.5

Supp. Fig. S12. **Rooted maximum-likelihood phylogeny of CSP1-4 and Limnocylindraceae families**. Evolutionary history constructed using the LG + C30 model, which combines the LG amino acid substitution model with a 30-profile mixture model to account for compositional heterogeneity. The analysis includes all publicly available reference genomes (GTDB R220 release) for both families, together with the 72 Chloroflexota MAGs recovered from the pdCEL database. The coloured strip represents the inferred habitat (i.e., soil, sediment, and freshwater) for each genome. Blue rings and circles denote SH-aLRT and UFBoot support values exceeding 80 and 95, respectively. The scale bar indicates the number of substitutions per site. Outgroup rooting was performed by using members of the P2-11E Chloroflexota order. Source data are provided as a Source Data file.

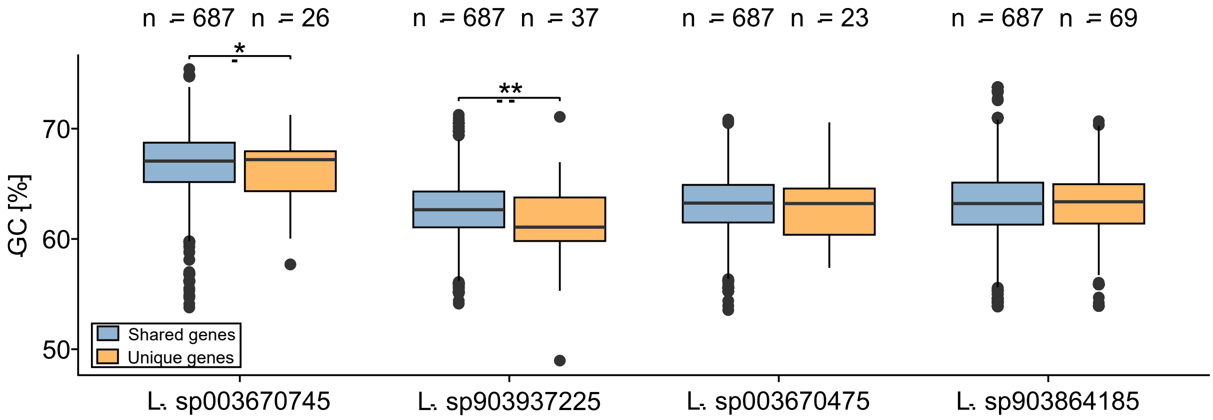

Supp. Fig. S13. GC content dynamics. GC content distribution between shared (blue) and unique (orange) orthologous genes across the four Limnocylindraceae species (i.e., *L. sp003670745*; *L. sp903937227*; *L. sp003670475*; *L. sp903864185*). Pairwise statistical differences are highlighted via the means of stars (*p < 0.05; **p < 0.01; ***p < 0.001). Star absence indicates non-significant results. The central line across the boxplots identifies the median, marking the dataset's midpoint. The box itself demarcates the interquartile range, extending from the first quartile to the third quartile, encapsulating the central 50% of the data. The whiskers project from the box to the furthest data points not categorized as outliers and show the spread of the main body of the dataset. All supplementary statistics are reported in Supp. Data S15. Source data are provided as a Source Data file.

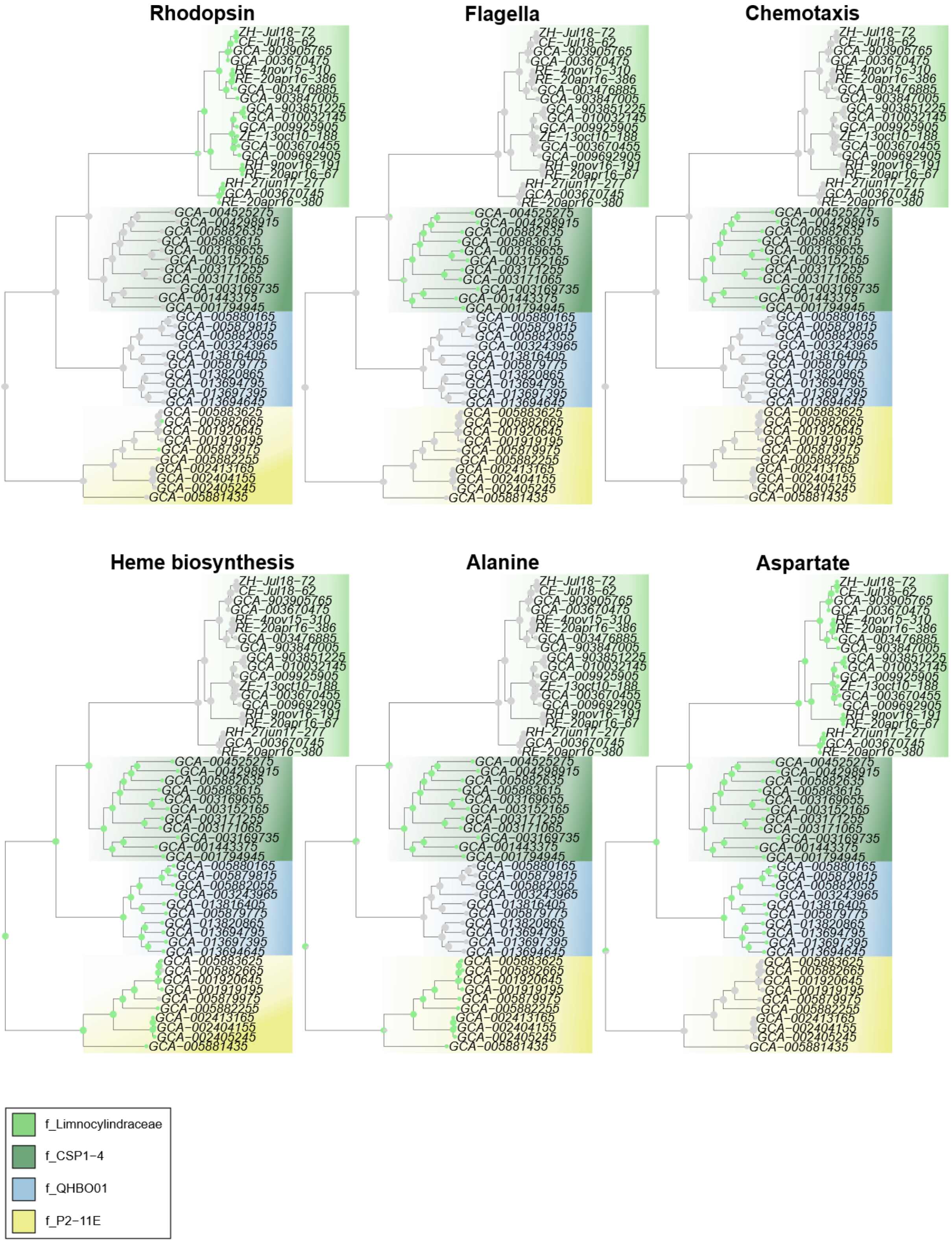
Rhodopsin
Flagella
Chemotaxis
Heme biosynthesis
Alanine
Aspartate
ZH-Jul18-72
CE-Jul18-62
GCA-903905765
GCA-003670475
RE-4nov15-310
RE-20apr16-386
GCA-003476885
GCA-903847005
GCA-903851225
GCA-010032145
GCA-009925905
ZE-13oct10-188
GCA-003670455
GCA-009692905
RH-9nov16-191
RE-20apr16-67
RH-27jun17-277
GCA-003670745
RE-20apr16-380
GCA-004525275
GCA-004298915
GCA-005882635
GCA-005883615
GCA-003169655
GCA-003152165
GCA-003171255
GCA-003171065
GCA-003169735
GCA-001443375
GCA-001794945
GCA-005880165
GCA-005879815
GCA-005882055
GCA-003243965
GCA-013816405
GCA-005879775
GCA-013820865
GCA-013694795
GCA-013697395
GCA-013694645
GCA-005883625
GCA-005882665
GCA-001920645
GCA-001919195
GCA-005879975
GCA-005882255
GCA-002413165
GCA-002404155
GCA-002405245
GCA-005881435
f_Limnocylindraceae
f_CSP1-4
f_QHBO01
f_P2-11E

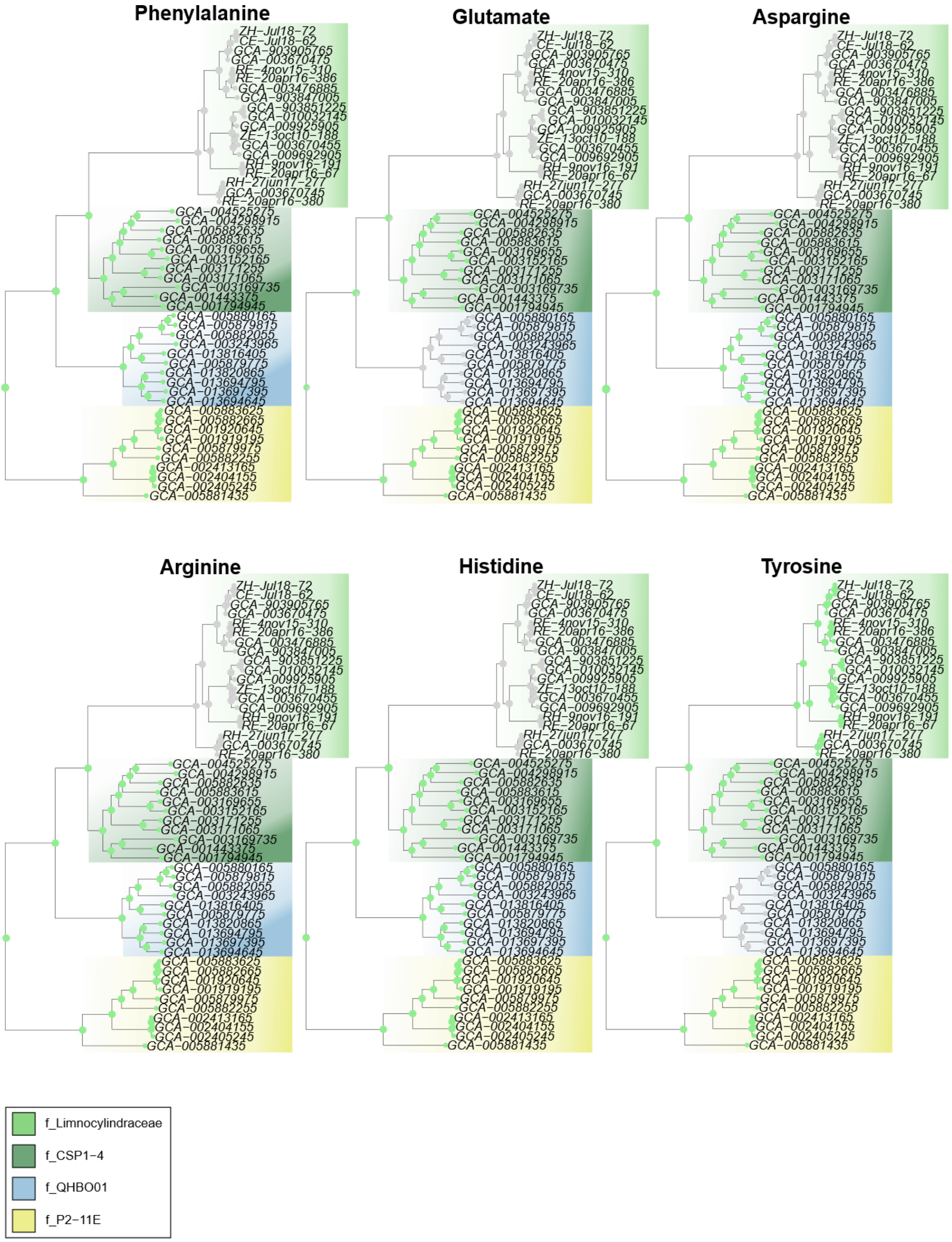
Phenylalanine
Glutamate
Aspargine
Arginine
Histidine
Tyrosine
f_Limnocylindraceae
f_CSP1−4
f_QHBO01
f_P2−11E

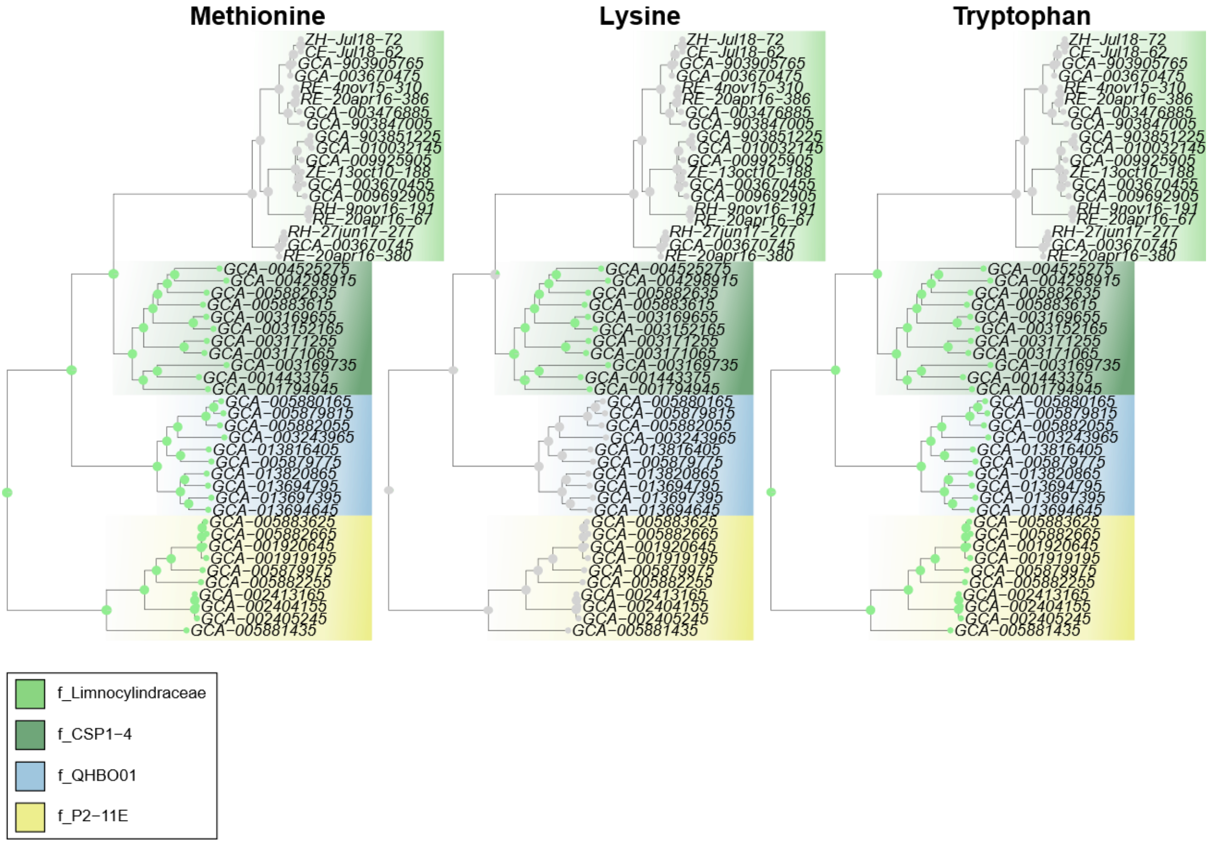

Supp. Fig. S14. Ancestral State reconstruction. The figure depicts the rooted maximum-likelihood phylogeny of the Limnocylindria class (Chloroflexota phylum) with ancestral states reconstructed separately for each trait (i.e., Rhodopsin, flagellar assembly, chemotaxis, heme biosynthesis, alanine, aspartate, glutamate, asparagine, arginine, histidine, tyrosine, methionine, lysine, tryptophan, and phenylalanine biosynthesis). Ancestral states were inferred under an equal-rates (ER) Mk model using 1’000 stochastic mappings per trait. Node pies indicate the posterior probability that the ancestral node possesses the trait, with green being present and grey being absent. Tip circles represent the observed state in each genome (same colour key as the nodes). Coloured boxes indicate the taxonomic affiliation: light green for Limnocylindraceae, dark green for CSP1-4, light blue for QHB01, and yellow for P2-11E. Source data are provided as a Source Data file.

References

1. Palmer, M. *et al*. Thermophilic Dehalococcoidia with unusual traits shed light on an unexpected past. *The ISME Journal 2023 17:7* 17, 952–966 (2023).

2. Bulzu, P.-A., Kavagutti, V. S., Andrei, A.-S. & Ghai, R. The Evolutionary Kaleidoscope of Rhodopsins. *mSystems* 7, (2022).
