# Supplementary material for: Deep-branching Chloroflexota lineages illuminate the eco-evolutionary foundation of cross-ecosystem colonization
Source: Nat Commun. 2026 Apr 1;17:4696. doi: 10.1038/s41467-026-71228-y (PMC13212565; doi:10.1038/s41467-026-71228-y)
Supplement: Supplementary file 2 — Description of Additional Supplementary Files [file 41467_2026_71228_MOESM2_ESM.pdf]

Description of Additional Supplementary Files

Supplementary Data S1

This sheet lists the 236 Chloroflexota genomes analyzed in this study, together with their accession numbers, taxonomic assignments, genomic features, and associated habitats.

Supplementary Data S2

This sheet lists the Chloroflexota genomes used for evolutionary history reconstruction, along with their accession numbers, taxonomy, and habitat of origin.

Supplementary Data S3

This sheet contains the list of Limnocylindrus species genomes used in this study, along with their accession numbers, taxonomic classification, genomic properties, and associated habitats.

Supplementary Data S4

This sheet lists the 118 TIGR marker gene IDs used for evolutionary history reconstruction in this study.

Supplementary Data S5

This sheet contains the Sandpiper results, reporting the abundance of Chloroflexota across simplified habitat categories.

Supplementary Data S6
This sheet presents the metabolic reconstruction of Chloroflexota genomes, focusing on transporter systems.

Supplementary Data S7
This sheet presents the metabolic reconstruction of Chloroflexota genomes related to amino acid biosynthesis pathways.

Supplementary Data S8
This sheet presents the percentage completeness of amino acid biosynthesis pathways in Chloroflexota genomes.

Supplementary Data S9
This sheet presents the metabolic reconstruction of Chloroflexota genomes related to carbohydrate metabolism, energy metabolism, and motility pathways.

Supplementary Data S10
This sheet presents the metabolic reconstruction of Chloroflexota genomes related to DNA repair mechanisms.

Supplementary Data S11
This sheet presents the metabolic reconstruction of CSP1–4 genomes, comparing representatives from soil, sediment, and freshwater habitats.

Supplementary Data S12
This sheet lists the Gammaproteobacteria genomes (family Methylophilaceae) used for nitrogen and carbon content analyses.

Supplementary Data S13
This sheet lists the Planctomycetota genomes (class Phycisphaerae) used for nitrogen and carbon content analyses.

Supplementary Data S14
This sheet contains the statistical analyses associated with the main figures.

Supplementary Data S15
This sheet contains the statistical analyses associated with the supplementary figures.
