# Supplementary material for: Deep-branching Chloroflexota lineages illuminate the eco-evolutionary foundation of cross-ecosystem colonization
Source: Nat Commun. 2026 Apr 1;17:4696. doi: 10.1038/s41467-026-71228-y (PMC13212565; doi:10.1038/s41467-026-71228-y)
Supplement: Supplementary file 4 — Reporting Summary [file 41467_2026_71228_MOESM4_ESM.pdf]

nature portfolio

Corresponding author(s): Adrian-Stefan Andrei

Last updated by author(s): Feb 6, 2026

Reporting Summary

Nature Portfolio wishes to improve the reproducibility of the work that we publish. This form provides structure for consistency and transparency in reporting. For further information on Nature Portfolio policies, see our Editorial Policies and the Editorial Policy Checklist.

Statistics

For all statistical analyses, confirm that the following items are present in the figure legend, table legend, main text, or Methods section.

n/a	Confirmed	
☐	☒	The exact sample size (*n*) for each experimental group/condition, given as a discrete number and unit of measurement
☒	☐	A statement on whether measurements were taken from distinct samples or whether the same sample was measured repeatedly
☐	☒	The statistical test(s) used AND whether they are one- or two-sided<br>*Only common tests should be described solely by name; describe more complex techniques in the Methods section.*
☒	☐	A description of all covariates tested
☐	☒	A description of any assumptions or corrections, such as tests of normality and adjustment for multiple comparisons
☐	☒	A full description of the statistical parameters including central tendency (e.g. means) or other basic estimates (e.g. regression coefficient) AND variation (e.g. standard deviation) or associated estimates of uncertainty (e.g. confidence intervals)
☐	☒	For null hypothesis testing, the test statistic (e.g. *F*, *t*, *r*) with confidence intervals, effect sizes, degrees of freedom and *P* value noted<br>*Give P values as exact values whenever suitable.*
☒	☐	For Bayesian analysis, information on the choice of priors and Markov chain Monte Carlo settings
☒	☐	For hierarchical and complex designs, identification of the appropriate level for tests and full reporting of outcomes
☐	☒	Estimates of effect sizes (e.g. Cohen's *d*, Pearson's *r*), indicating how they were calculated

Our web collection on statistics for biologists contains articles on many of the points above.

Software and code

Policy information about availability of computer code

Data collection

FTP links for genomic data were constructed based on the GenBank assembly summary file from NCBI. Briefly, the GenBank assembly summary file was downloaded by using the command wget ftp://ftp.ncbi.nlm.nih.gov/genomes/ASSEMBLY_REPORTS/assembly_summary_genbank.txt. Target genome accessions from GTDB were then searched within this file, and the matches were saved as a list. Using an awk command (awk 'BEGIN{FS=OFS="/";filesuffix="genomic.fna.gz"}{ftpdir=$0;asm=$10;file=asm"_"filesuffix;print "wget "ftpdir,file}' list.txt > download_genomes.sh), the FTP directory and accession were extracted from relevant fields in the list and formatted into wget commands to download the genomic fna.gz files. The generated script, download_genomes.sh, was then executed to download the target genomes.

Data analysis

GTDB-Tk v2.4.0
PRODIGAL v2.6.3
HMMER hmmscan v3.1b2
PRANK v170427
BMGE v1.12
IQ-TREE v2.0.6
FAMSA v1.2
SingleM v0.20.3
pfam_scan.pl
InterProScan v5.24-63.0
BlastKOALA v3.1
PRANK v170427
IQ-TREE v2.1.3

phytools v2.0
HYPHY v2.5.32
seqstat v1.9g
Bio::SeqIO
R v4.2.2
RStudio v1.3.1093
NIS-Elements Advanced Research software v5.01.00
The source code generated in this study is publicly available on GitHub at https://github.com/MiELevog/Cross-ecosystem-colonization

Public databases utilised in this study:
GTDB R09-RS220
pdCEL v1.0
TIGRFAMs v15.0
Sandpiper v0.3.0
CDD v3.14
SMART v7.1
HAMAP v201701.18
Pfam release 32
COG
KEGG

For manuscripts utilizing custom algorithms or software that are central to the research but not yet described in published literature, software must be made available to editors and reviewers. We strongly encourage code deposition in a community repository (e.g. GitHub). See the Nature Portfolio guidelines for submitting code & software for further information.

Data

Policy information about availability of data

All manuscripts must include a data availability statement. This statement should provide the following information, where applicable:

- Accession codes, unique identifiers, or web links for publicly available datasets
- A description of any restrictions on data availability
- For clinical datasets or third party data, please ensure that the statement adheres to our policy

Data availability
All genomic data utilized in this study is publicly available. 236 MAG IDs and their NCBI accession numbers are provided in Supp. Data S1. NCBI SRA IDs are provided for 14,644 metagenomes in Supp. Data S5. All additional important data supporting the study's conclusions are included in this publication and its Supp. Data 2–15. Source data are provided with this paper.
Genome accession numbers:
GCA_947441585.1 GCA_947442125.1 GCA_947442155.1 GCA_947444015.1 GCA_947445235.1 GCA_001443375.1 GCA_001794945.1 GCA_001795245.1
GCA_001795345.1 GCA_001797125.1 GCA_001917735.1 GCA_001919195.1 GCA_001920645.1 GCA_002404035.1 GCA_002404155.1 GCA_002405245.1
GCA_002413045.1 GCA_002413165.1 GCA_002427725.1 GCA_003131785.1 GCA_003131865.1 GCA_003137015.1 GCA_003141245.1 GCA_003141455.1
GCA_003141955.1 GCA_003142555.1 GCA_003142735.1 GCA_003142755.1 GCA_003152165.1 GCA_003152995.1 GCA_003153755.1 GCA_003154695.1
GCA_003154815.1 GCA_003155435.1 GCA_003155555.1 GCA_003156975.1 GCA_003156995.1 GCA_003157175.1 GCA_003157855.1 GCA_003157875.1
GCA_003157935.1 GCA_003158295.1 GCA_003158495.1 GCA_003158555.1 GCA_003158575.1 GCA_003158675.1 GCA_003158875.1 GCA_003162565.1
GCA_003162715.1 GCA_003165505.1 GCA_003165675.1 GCA_003165825.1 GCA_003167665.1 GCA_003169285.1 GCA_003169655.1 GCA_003169735.1
GCA_003170695.1 GCA_003171035.1 GCA_003171065.1 GCA_003171255.1 GCA_003171455.1 GCA_003243965.1 GCA_003247655.1 GCA_003250155.1
GCA_003476885.1 GCA_003670455.1 GCA_003670475.1 GCA_003670745.1 GCA_004297395.1 GCA_004298915.1 GCA_004525275.1 GCA_005879625.1
GCA_005879775.1 GCA_005879815.1 GCA_005879975.1 GCA_005880035.1 GCA_005880165.1 GCA_005880215.1 GCA_005880335.1 GCA_005880575.1
GCA_005880715.1 GCA_005880805.1 GCA_005880935.1 GCA_005881055.1 GCA_005881085.1 GCA_005881435.1 GCA_005881735.1 GCA_005881885.1
GCA_005881975.1 GCA_005882055.1 GCA_005882255.1 GCA_005882635.1 GCA_005882665.1 GCA_005882765.1 GCA_005883475.1 GCA_005883505.1
GCA_005883525.1 GCA_005883615.1 GCA_005883625.1 GCA_005883775.1 GCA_005883785.1 GCA_005884355.1 GCA_005887995.1 GCA_005888015.1
GCA_005889075.1 GCA_005889275.1 GCA_005889295.1 GCA_005889405.1 GCA_005889415.1 GCA_005889445.1 GCA_005889475.1 GCA_009692785.1
GCA_009692905.1 GCA_009919005.1 GCA_009919365.1 GCA_009925905.1 GCA_010032145.1 GCA_012026785.1 GCA_013694645.1 GCA_013694795.1
GCA_013694835.1 GCA_013695255.1 GCA_013695505.1 GCA_013695575.1 GCA_013696275.1 GCA_013696945.1 GCA_013697395.1 GCA_013698095.1
GCA_013698385.1 GCA_013813905.1 GCA_013815585.1 GCA_013816405.1 GCA_013820865.1 GCA_013822395.1 GCA_903820655.1 GCA_903822495.1
GCA_903825635.1 GCA_903833645.1 GCA_903847005.1 GCA_903851225.1 GCA_903859655.1 GCA_903864185.1 GCA_903874285.1 GCA_903891385.1
GCA_903894305.1 GCA_903901465.1 GCA_903905765.1 GCA_903914395.1 GCA_903917495.1 GCA_903924005.1 GCA_903925955.1 GCA_903928275.1
GCA_903928775.1 GCA_903929625.1 GCA_903930745.1 GCA_903931025.1 GCA_903933435.1 GCA_903934305.1 GCA_903935445.1 GCA_903936785.1
GCA_903937225.1 GCA_903938075.1 GCA_903940915.1 GCA_903944295.1 GCA_903952625.1 GCA_903953035.1 GCA_903958405.1 GCA_903959635.1
GCA_903961065.1 GCA_947455735.1 GCA_947456105.1 GCA_947457755.1 GCA_947458195.1 GCA_947459615.1 GCA_947460315.1 GCA_947461465.1
GCA_947461955.1 GCA_947462305.1 GCA_947462545.1 GCA_947463525.1 GCA_947463875.1 GCA_947464285.1 GCA_947464535.1 GCA_947465175.1
GCA_947465195.1 GCA_947465455.1 GCA_947466305.1 GCA_947466975.1 GCA_947468805.1 GCA_947469145.1 GCA_947469315.1 GCA_947469505.1
GCA_947470425.1 GCA_947470485.1 GCA_947470785.1 GCA_947471065.1 GCA_947472105.1 GCA_947472345.1 GCA_947472375.1 GCA_947472415.1
GCA_947472525.1 GCA_947472805.1 GCA_947473115.1 GCA_947473845.1 GCA_947473995.1 GCA_947475545.1 GCA_947476265.1 GCA_947477135.1
GCA_947477255.1 GCA_947479575.1 GCA_947479725.1 GCA_947479875.1 GCA_947479885.1 GCA_947480455.1 GCA_947480535.1 GCA_947481995.1
GCA_947482045.1 GCA_947482475.1 GCA_947482765.1 GCA_947483025.1 GCA_947484395.1 GCA_947485065.1 GCA_947486925.1 GCA_947490635.1
GCA_947490685.1 GCA_947491145.1 GCA_947491345.1 GCA_947499445.1 GCA_947501155.1 GCA_947501735.1 GCA_947502115.1 GCA_947505265.1
GCA_947505545.1 GCA_947505715.1 GCA_947507665.1 GCA_947507995.1

Research involving human participants, their data, or biological material

Policy information about studies with human participants or human data. See also policy information about sex, gender (identity/presentation), and sexual orientation and race, ethnicity and racism.

Reporting on sex and gender	This study does not involve human research participants.
Reporting on race, ethnicity, or other socially relevant groupings	This study does not involve human research participants.
Population characteristics	This study does not involve human research participants.
Recruitment	This study does not involve human research participants.
Ethics oversight	This study does not involve human research participants.

Note that full information on the approval of the study protocol must also be provided in the manuscript.

Field-specific reporting

Please select the one below that is the best fit for your research. If you are not sure, read the appropriate sections before making your selection.

☐ Life sciences ☐ Behavioural & social sciences ☒ Ecological, evolutionary & environmental sciences

For a reference copy of the document with all sections, see nature.com/documents/nr-reporting-summary-flat.pdf

Ecological, evolutionary & environmental sciences study design

All studies must disclose on these points even when the disclosure is negative.

Study description	This is an exploratory genome-resolved metagenomic study focused on the analysis of environmental bacterial genomes. The nature of the study does not necessitate any treatment factors, interactions, design structure (factorial, nested, hierarchical) or replicates.
Research sample	This study involved the analysis of environmentally recovered genome-resolved metagenomic data. A part of the data is derived from the pdCEL database: https://doi.org/10.1038/s41467-024-47767-7. The pdCEL database contains approximately 5,500 high-quality MAGs sourced from Central European freshwater lakes with varying trophic statuses. These MAGs were subjected to meticulous curation, including taxonomic validation, checks for GC content consistency, and the application of stringent thresholds for genome completeness and contamination to ensure data integrity. From this reclassified dataset, we identified 72 Limnocylindria MAGs based on the updated taxonomy. To expand our dataset further, we incorporated an additional 164 MAGs indexed in the Genome Taxonomy Database (GTDB), resulting in a curated collection of 236 high-quality MAGs.
Sampling strategy	The genomic data analyzed in this study were derived from our previously published pdCEL metagenomic sampling campaign and additional publicly available genomes from GTDB. In the pdCEL study, freshwater samples were collected from five Central European lakes (Czech Republic and Switzerland), including both epilimnetic and hypolimnetic layers. Sample size in the present study was determined by the number of metagenome-assembled genomes (MAGs) that could be recovered. No a priori sample size or power calculation was performed, because sample availability was constrained by genome recovery and quality control rather than experimental replication.
Data collection	The genomic data analyzed in this study is derived from previously published metagenomic datasets (pdCEL) generated by our group, and were not recorded manually in the present work. The original pdCEL study describes the collection of freshwater samples from five lakes spanning the Czech Republic and Switzerland, including both epilimnetic and hypolimnetic samples. In that study, water samples were collected using a Friedinger sampler and processed by sequential peristaltic filtration through polycarbonate membrane filters. DNA was extracted from the 0.22-µm filters using the ZR Soil Microbe DNA MiniPrep kit, quantified using the Qubit dsDNA BR assay on a Qubit 2.0 fluorometer, and assessed for integrity by agarose gel electrophoresis. Shotgun metagenomic sequencing was performed on an Illumina NovaSeq 6000 platform (2×150 bp). In the present study, all downstream genomic features and metadata were generated computationally by the authors using standardized bioinformatic workflows and recorded directly from pipeline outputs rather than through manual transcription.
Timing and spatial scale	The metagenomic data analyzed here were generated in our previously published pdCEL study. In that study, sampling was conducted across multiple cohorts spanning 2013–2019, including June 2015–August 2017 (Římov Reservoir and Jiřická pond), 2013–2019, 2025 (Lake Zurich), June 2018 (Lake Thun), and July and October 2018 (Lake Constance).
Data exclusions	No data were excluded from the analyses.
Reproducibility	The data generated are available in referenced public repositories, ensuring transparency and accessibility. All methods used in this study are extensively cited, and the parameters for software applications are fully documented. Given the study's reliance on environmental samples, no attempts were made to replicate the experiments. Covariates were not explicitly controlled in this study, as our analyses were primarily comparative and genome-based using curated MAG-derived features.

Randomization	This is an exploratory study and randomization is not relevant to the study design.
Blinding	Blinding was not performed because it was not relevant to this study. This study was an exploratory survey of microbial diversity without prior expectations that would influence the analyses.

Did the study involve field work? ☒ Yes ☐ No

Field work, collection and transport

Field conditions	CARD-FISH. Field conditions at the sampling site were recorded on 31 March 2021. Water clarity was 3 m Secchi depth. At 5 m depth, temperature was 6.6 °C, conductivity 302 µS cm^{-1}, dissolved oxygen 14 mg L^{-1}, turbidity 1.5 NTU, and chlorophyll a 17.4 µg L^{-1}.
Location	Lake Zurich (406m a.s.l., 47°18′N, 8°34′E, Switzerland); 5 m depth.
Access & import/export	All samples were collected in compliance with local, cantonal, and national laws. No permits were required for lake sampling. Sampling at Lake Zurich was conducted by the authors at the University of Zurich's Limnological Station.
Disturbance	No disturbances were caused by the sampling procedures.

Reporting for specific materials, systems and methods

We require information from authors about some types of materials, experimental systems and methods used in many studies. Here, indicate whether each material, system or method listed is relevant to your study. If you are not sure if a list item applies to your research, read the appropriate section before selecting a response.

Materials & experimental systems

n/a	Involved in the study
☒	☐ Antibodies
☒	☐ Eukaryotic cell lines
☒	☐ Palaeontology and archaeology
☒	☐ Animals and other organisms
☒	☐ Clinical data
☒	☐ Dual use research of concern
☒	☐ Plants

Methods

n/a	Involved in the study
☒	☐ ChIP-seq
☒	☐ Flow cytometry
☒	☐ MRI-based neuroimaging

Plants

Seed stocks	Not applicable.
Novel plant genotypes	Not applicable.
Authentication	Not applicable.
